# Identification of Abscisic Acid-Dependent Phosphorylated Basic Helix-Loop-Helix Transcription Factors in Guard Cells of *Vicia faba* by Mass Spectrometry

**DOI:** 10.3389/fpls.2021.735271

**Published:** 2021-12-20

**Authors:** Yuki Hayashi, Yohei Takahashi, Kohei Fukatsu, Yasuomi Tada, Koji Takahashi, Keiko Kuwata, Takamasa Suzuki, Toshinori Kinoshita

**Affiliations:** ^1^Division of Biological Science, Graduate School of Science, Nagoya University, Nagoya, Japan; ^2^Cell and Developmental Biology Section, Division of Biological Sciences, University of California San Diego, San Diego, CA, United States; ^3^Institute of Transformative Bio-Molecules (WPI-ITbM), Nagoya University, Nagoya, Japan; ^4^Center for Gene Research, Nagoya University, Nagoya, Japan; ^5^Department of Biological Chemistry, College of Bioscience and Biotechnology, Chubu University, Kasugai, Japan

**Keywords:** abscisic acid, Arabidopsis, bHLH transcription factor, protein phosphorylation, stomata, *Vicia faba*, 14-3-3 protein, mass spectrometry

## Abstract

An unknown 61 kDa protein is phosphorylated by abscisic acid (ABA)-activated protein kinase in response to ABA and binds to 14-3-3 protein in a phosphorylation-dependent manner in guard-cell protoplasts (GCPs) from *Vicia faba*. Subsequently, ABA-dependent phosphorylated proteins were identified as basic helix–loop–helix transcription factors, named ABA-responsive kinase substrates (AKSs) in GCPs from *Arabidopsis thaliana*. However, whether the 61 kDa protein in *Vicia* GCPs is an AKS is unclear. We performed immunoprecipitation of ABA-treated *Vicia* GCPs using anti-14-3-3 protein antibodies and identified several AKS isoforms in *V. faba* (VfAKSs) by mass spectrometry. The 61 kDa protein was identified as VfAKS1. Phosphoproteomic analysis revealed that VfAKSs are phosphorylated at Ser residues, which are important for 14-3-3 protein binding and monomerisation, in response to ABA in GCPs. Orthologs of AtABCG40, an ABA importer in guard cells, and CHC1, a clathrin heavy chain and a regulator of stomatal movement, also co-immunoprecipitated with 14-3-3 protein from guard cells.

## Introduction

Stomata in plant epidermis consist of a pair of guard cells. The stomatal aperture is regulated by environmental signals—such as light, drought and CO_2_—and controls CO_2_ uptake for photosynthesis and transpiration (Shimazaki et al., 2007; Inoue and Kinoshita 2017). The plant hormone abscisic acid (ABA) promotes plant adaptation to drought stress by regulating gene expression, ion transport and enzyme activities (Cutler et al., 2010; Dejonghe et al., 2018). ABA induces stomatal closure to prevent water loss by transpiration (Kim et al., 2010; Munemasa et al., 2015). Analysis of the core complex of the ABA-dependent early signalling pathway showed that the ABA receptor PYRABACTIN RESISTANCE/PYRABACTIN RESISTANCE-LIKE/REGULATORY COMPONENTS OF ABA RECEPTOR (PYR/PYL/RCAR) inhibits type 2C protein phosphatases (PP2Cs), which negatively regulate ABA signalling in the core complex in response to ABA (Ma et al., 2009; Melcher et al., 2009; Miyazono et al., 2009; Nishimura et al., 2009; Park et al., 2009). SNF1-related protein kinases 2 (SnRK2s) are activated by inhibition of PP2C and phosphorylate their substrates, such as the SLOW ANION CHANNEL-ASSOCIATED 1 anion channel in the plasma membrane, which is important for stomatal closure, and basic region/Leu zipper motif transcription factors of ABA-responsive element (ABRE)-binding proteins (AREBs)/ABRE-binding factors (Yoshida et al., 2006; Fujii et al., 2009; Geiger et al., 2009; Lee et al., 2009; Umezawa et al., 2009; Vlad et al., 2009; Kline et al., 2010).

It has been demonstrated that protein phosphorylation and phosphorylation-dependent binding of 14-3-3 protein have important roles for stomatal movements (Zhang et al., 2014; Cotelle and Leonhardt, 2016). A 61 kDa protein is phosphorylated in response to ABA according to protein-blot analysis using recombinant glutathione S-transferase (GST)-14-3-3 protein as the probe. The 61 kDa protein binds to 14-3-3 protein in a phosphorylation-dependent manner in guard-cell protoplasts (GCPs) from *V. faba* (Takahashi et al., 2007). Further characterisation suggested that the 61 kDa protein is soluble and phosphorylated by ABA-activated protein kinase (AAPK; Li and Assmann, 1996; Li et al., 2000; Takahashi et al., 2007). However, the gene encoding the 61 kDa protein is unknown.

Using a similar approach, three ABA-dependent phosphorylated proteins were found in Arabidopsis guard cells and were identified by mass spectrometry (MS) as basic helix–loop–helix (bHLH) transcription factors, named ABA-responsive kinase substrate (AKS) 1 to AKS3 (Takahashi et al., 2013). AKSs possess two 14-3-3 protein-binding sites in which Ser residues (Ser-30 and -157 in AKS1) are phosphorylated in response to ABA, resulting in binding of 14-3-3 protein. AKSs facilitate stomatal opening by stimulating transcription of genes encoding K^+^_in_ channels, including *KAT1*, in guard cells by binding to their promoter regions, and their function is counteracted by ABA-dependent phosphorylation of the AKS proteins downstream of the PYR/PYL/RCAR ABA receptor (Takahashi et al., 2013). AKS1 is likely to bind to DNA as a dimer, and AKS1 dimer formation is reportedly disrupted by phosphorylation at Ser-284, -288 and-290 in AKS1 (Takahashi et al., 2016). AKSs are phosphorylated by SnRK2 kinases in response to ABA *in vivo*. (Takahashi et al., 2017). Furthermore, ABA-induced AKS1 monomerisation and detachment from the *KAT1* promoter have been reconstituted *in vitro* using recombinant PYR1 ABA receptor, HAB1 PP2C, and OST1/SnRK2.6 protein-kinase proteins, suggesting the minimal signalling mechanism from the ABA receptor to DNA (Takahashi et al., 2017). Therefore, AKSs play pivotal roles in ABA-induced repression of potassium-channel gene expression in Arabidopsis guard cells.

The *Vicia* 61 kDa protein shows similar properties to Arabidopsis AKS proteins, including ABA-induced phosphorylation and 14-3-3 protein binding (Takahashi et al., 2007). However, the 61 kDa protein in *Vicia* GCPs has not been identified because of the difficulty identifying *V. faba* proteins by mass spectrometry. To overcome this issue, we constructed an RNA-sequencing (RNA-seq) expression database of *V. faba* for identification of proteins. We subsequently purified and identified the 61 kDa protein as a bHLH transcription factor of *V. faba* (VfAKS) by MS. Furthermore, we mapped *in vivo* phosphorylation sites in VfAKS proteins in response to ABA using 1 mg of protein from *Vicia* GCPs.

## Materials and Methods

### Plant Materials and Isolation of GCPs

*Vicia faba* (Broad bean; Ryosai Issun) was cultivated hydroponically in a greenhouse as described previously (Shimazaki et al., 1992). GCPs were isolated enzymatically from lower epidermis of 4- to 6-week-old leaves as described elsewhere (Kinoshita and Shimazaki, 1999). Protein concentrations were determined using a Bradford kit according to the manufacturer’s instructions (Bio-Rad Laboratories, Hercules, CA).

### Protein Blotting With a Glutathione S-Transferase–14-3-3 Protein and Immunoblotting

Protein-blot analysis was performed using a GST–GF14phi fusion protein as described (Takahashi et al., 2007; Minami et al., 2019). GF14phi is an Arabidopsis 14-3-3 protein (Kinoshita and Shimazaki, 1999). Immunoblotting was performed using an antibody for *Vicia* 14-3-3 protein (Vf14-3-3a) as described previously (Emi et al., 2001; Takahashi et al., 2007).

### Co-immunoprecipitation of 14-3-3 Binding Protein and Liquid Chromatography–Tandem Mass Spectrometry

To purify 14-3-3 binding proteins, GCPs from *V. faba* (330–500 μg) in suspension buffer (5 mM MES-NaOH [pH 6.0], 10 mM KCl, 0.4 M mannitol and 1 mM CaCl_2_) were treated with 10 μM ABA for 10 min in the dark. The 14-3-3 binding proteins were co-immunoprecipitated from the supernatant using an antibody against vf14-3-3a (Kinoshita and Shimazaki, 1999; Takahashi et al., 2013). The precipitated proteins were separated by sodium dodecyl sulphate-polyacrylamide gel electrophoresis (SDS-PAGE). The sample lanes were excised into 7 to 10 segments and subsequently subjected to in-gel digestion according to previous method (Shevchenko et al., 2006). The digested peptides were analysed by nano-liquid chromatography–tandem mass spectrometry (LC-MS/MS). Nano-LC-MS/MS was performed using a Dionex U3000 Gradient Pump (Thermo Fisher Scientific) connected to a Q-Exactive Hybrid Quadrupole-Orbitrap Mass Spectrometer (Thermo Fisher Scientific). Peptides were loaded into a trap column [L-column ODS (300 μM internal diameter [ID] × 5 mM, 5 μM particle size), CERI] and separated at 500 nl/min using a 5–40% buffer B gradient over 100 min on a nano-HPLC capillary column [NTCC-360 (100 μM I.D. × 125 mM, 3 μM particle size), Nikkyo Technos]. The composition of the LC buffer A was 0.5% (v/v) acetic acid in water and LC buffer B was of 80% (v/v) acetonitrile, 0.5% (v/v) acetic acid. The Xcalibur 3.0.63 system (Thermo) was used to record peptide spectra over the mass range of m/z 350–1800 (70,000 resolution, 3e6 AGC, 60 ms injection time), followed by ten data-dependent high-energy collisional dissociations (HCD) MS/MS spectra generated from ten highest-intensity precursor ions (17,500 resolution, 1e5 AGC, 60 ms injection time, 27 NCE).

Peptides and proteins were identified by means of automated database searching using Proteome Discoverer v. 2.2.0.388 (Thermo Fisher Scientific) against the *V. faba* expression database. The following search parameters were employed as: peptide mass range (m/z), 350–1,800 Da; enzyme specificity, trypsin or LysC with up to two missed cleavages; and precursor ion and peptide fragment mass tolerances, ± 10 ppm and ± 0.02 Da, respectively. Peptide validation was performed using the Percolator algorithm, and only high-confidence peptides were used for peptide identification and quantification. The identified peptides in each sample lane were summarised (Table 1).

**Table 1:** List of bHLH DNA-binding superfamily proteins in Vicia GCPs.

Accession	Protein length (aa)	Estimated molecular mass (kDa)	14–3-3 IP (PSMs)						
			Experiment 1		Experiment 2		Experiment 3		Number of unique peptides
			−ABA	+ABA	−ABA	+ABA	−ABA	+ABA	
VfAKS1	423	47.7	–	37	3	32	–	20	5
VfAKS2	442	48.0	–	12	1	8	–	7	1
VfAKS3	384	41.9	–	3	–	3	–	6	2
VfAKS4	326	35.4	2	22	4	22	–	13	6
VfAKS5	190	21.5	–	2	–	4	–	–	1
VfAKS6	376	42.5	–	–	–	–	–	–	–
VfAKS7	345	39.2	–	–	–	–	–	–	–

*Protein length (number of amino acid) and estimated molecular mass of VfAKSs are listed. Peptide spectrum matches (PSMs), the total number of identified peptide spectra matched for the protein, of each protein in the immunoprecipitation using the antibody against Vicia 14–3-3a are indicated. –: not detected. Number of unique peptides from each VfAKSs in the immunoprecipitation of Experiment 1 is listed*.

### RNA-Seq Analysis and Construction of an Expression Database of *Vicia faba*

Total RNA was extracted from GCPs and leaves of 4- to 6-week-old *V. faba* using a TRIzol Plus RNA Purification Kit (Thermo Fisher Scientific). Three independent biological samples of each tissue type were harvested and snap-frozen in liquid nitrogen. Complementary DNA (cDNA) libraries were constructed from 1 μg of total RNA using the TruSeq RNA Sample Prep Kit v. 2 (Illumina) and sequenced on an NextSeq 500 (Illumina), yielding 13.0 to 19.8 million paired-end sequence reads per sample. Adapter sequences were trimmed with bcl2fastq2 (Illumina) and bases with low-quality scores were masked by N with the original script. In total, 288 million reads, which contained >50 non-masked bases, were used for *de novo* assembly by Trinity (Grabherr et al., 2011), yielding 134,130 contigs. The contigs were functionally annotated by BLASTX analysis against an Arabidopsis database (TAIR10). For MS determination of the amino acid sequences, these contigs were translated in six-frame and used for reference. To calculate expression levels, filtered reads were mapped to these contigs by Bowtie (Langmead et al., 2009).

### *In vitro* Translation of *VfAKSs*

*In vitro* transcription and translation were performed as described previously (Nomoto and Tada, 2018). First-strand cDNA was synthesised from total RNA of GCPs using SuperScript II reverse transcriptase (Invitrogen, Carlsbad, CA) with oligo(dT)_12–18_ as the primer and was used as the template for *in vitro* transcription. The PCR products were sequenced. To attach an N-terminal FLAG tag to the coding sequences of VfAKSs (VfAKS1-3), two-step PCR was carried out using the primers listed in Supplementary Table S2. *In vitro* transcription was carried out using a transcription kit (NUProtein). For *in vitro* translation, the resulting RNA solutions were mixed with wheat germ extract and amino acid mix (NUProtein) and incubated at 16°C for 10 h. The synthesised proteins were solubilised and separated by SDS-PAGE and detected by immunoblotting using an anti-FLAG antibody (Sigma).

### Phosphoproteomic Analysis of GCPs

GCPs from *V. faba* (1.3–1.6 mg of proteins) in suspension buffer were treated with 10 μM ABA for 10 min in the dark. The GCPs were disrupted by addition of trichloroacetic acid to a final concentration of 20% (v/v), followed by centrifugation. The precipitated guard-cell proteins were suspended in digestion buffer (8 M urea, 250 mM ammonium bicarbonate, 1× PhosSTOP [Roche]). The suspensions were reduced with Tris (2-carboxyethyl) phosphine hydrochloride, alkylated by iodoacetamide and digested with LysC (FUJIFILM), followed by tryptic digestion. Digestions were performed with the enhancer ProteaseMAX™ Surfactant (Promega). The digested samples were acidified and desalted on MonoSpin C18 columns (GL Sciences). Phosphopeptides were enriched by IMAC (Agilent) from 100 μg of digested peptides and diluted with 0.1% (v/v) TFA, 2% (v/v) AcCN in distilled water for nano-LC–MS/MS. Nano-LC–MS/MS was performed as described above.

Peptides and proteins were identified by means of automated database searching using Proteome Discoverer 2.2.0.388 (Thermo Fisher Scientific) against an expression database of *V. faba.* The following search parameters were employed as: peptide mass range (m/z), 350–1,800 Da; enzyme specificity, trypsin or LysC with up to two missed cleavages; precursor ion and peptide fragment mass tolerances, ± 10 ppm and ± 0.02 Da, respectively; static modification, carbamidomethyl (Cys); and dynamic modifications, phosphorylation (Ser, Thr and Tyr) and oxidation (Met). Peptide validation was performed using the Percolator algorithm, and only high-confidence peptides were used for peptide identification and quantification. The resulting dataset, which included information on annotated sequences, modifications, master protein accession, peptide spectrum matches (PSMs) and the total number of identified peptide spectra, for each identified peptide, was imported into Microsoft Excel. Using the filter function of Excel, peptides with no phosphorylated residues were excluded from the list. PSMs, the total number of identified peptide spectra matched for the protein, of each protein were compared between the datasets.

### Accession Numbers

Sequence data can be found in the Arabidopsis genome database TAIR10 under the following accession numbers: AKS1 (AT1G51140.1), AKS2 (AT1G05805.1), AKS3 (AT2G42280.1), AKS4 (AT2G43140.1), AKS5 (AT4G09180.1), AKS6 (AT1G35460.1), AtABCG40 (AT1G15520) and CHC1 (AT3G11130).

### Data Availability

RNA-seq data supporting the findings of this work have been deposited in the DNA Data Bank of Japan (DDBJ) under accession number DRA012337. The raw MS data have been deposited in the ProteomeXchange Consortium *via* the PRIDE partner repository under accession numbers, PXD027057 for immunoprecipitation and PXD027058 for phosphoproteomics.

## Results

### The 61 kDa Protein Bound to the 14-3-3 Protein From Guard Cells

**Figure 1:**
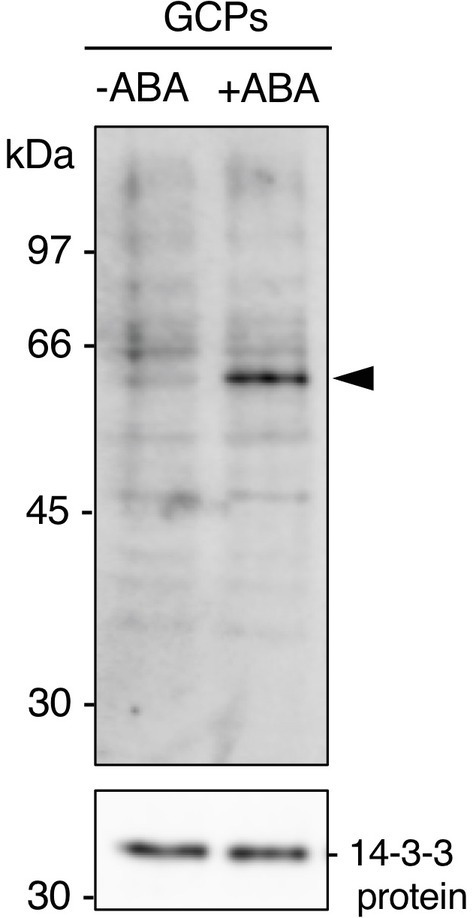
Detection of the 61 kDa protein in guard cell protoplasts (GCPs) from *Vicia faba* in response to ABA. ABA-dependent binding of 14-3-3 protein to the 61 kDa protein. GCPs were treated with 0.5% dimethyl sulphoxide as a solvent (−ABA) or 10 μM ABA (+ABA) and 20 μg of guard-cell proteins were separated by sodium dodecyl sulphate-polyacrylamide gel electrophoresis. Upper panel: 61 kDa protein detected by protein blotting using GST-14-3-3 protein as the probe. Lower panel: 14-3-3 protein detected by immunoblotting with an anti-14-3-3 antibody as the loading control. Arrowhead, the 61 kDa protein. Numbers at left indicate molecular weight markers.

GCPs from *V. faba* were treated with ABA at 10 μM for 10 min in the dark. A 61 kDa protein was confirmed as a 14-3-3 protein-binding protein in response to ABA by protein blot using a recombinant GST-14-3-3 fusion protein (Figure 1; Takahashi et al., 2007). GST alone did not show a prominent 61 kDa band (Kinoshita et al., 2003; Takahashi et al., 2007). Therefore, 14-3-3 protein specifically binds to the 61 kDa protein in an ABA-dependent manner.

### AKS Orthologs in *V. faba*

**Figure 2:**
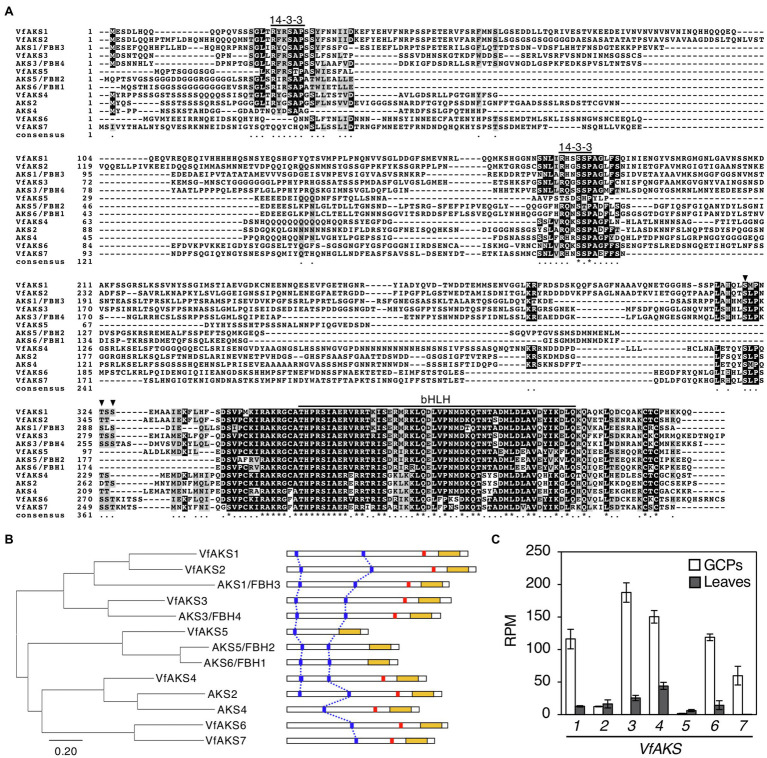
Evolutionary analysis of VfAKSs and AtAKSs. (A), Alignment of AKSs from *Vicia faba* (VfAKS1–VfAKS7) and *Arabidopsis thaliana* (AKS1–AKS6) generated using ClustalW (Thompson et al., 1994) with manual modification. Black boxes indicate highly conserved residues. Grey boxes indicate conservative residues. Consensus symbols “^*^” and “.” indicate perfect alignment and a site belonging to a group exhibiting weak similarity, respectively. Binding motifs for 14-3-3 protein (RXXpSXP) and bHLH motifs are indicated by lines. Phosphorylation sites that induce monomerisation of AKS1 and inhibit the transactivation activity of AKS1 are indicated by arrowheads. Dashes indicate gaps introduced to enable optimal sequence alignment. **(B)**, Phylogenetic tree of AKSs from *V. faba* and *A. thaliana.* Alignment for the phylogenetic tree was performed as described in **(A)**. The phylogenetic tree with the highest log likelihood (9768.99) was created by the maximum-likelihood method and JTT matrix-based model with MEGAX software. Structures of the AKS proteins are shown at right. 14-3-3 protein-binding motifs (blue lines), phosphorylation sites leading to inhibition of monomerisation (red lines) and bHLH domains (yellow boxes) are indicated. **(C)**, Expression levels of VfAKSs assayed by RNA-seq in GCPs and leaves. RPM values of VfAKS1–VfAKS7 are shown (*n* = 3, means ± SD).

In Arabidopsis, ABA-dependent phosphorylated proteins were identified as bHLH transcription factors, named ABA-responsive kinase substrate 1 (AKS1) to AKS3 in GCPs (Takahashi et al., 2013). AKSs possess two 14-3-3 protein-binding sites. However, there is no sequence information regarding AKSs in *V. faba*. Therefore, we performed RNA-seq analysis using GCPs and leaves from *V. faba* and constructed an expression database of *V. faba* by *de novo* assembly. At least seven AKS-like proteins, which possessed 14-3-3 binding sites and a bHLH domain, as well as Arabidopsis AKSs were expressed in *V. faba*. We named these proteins VfAKS1 to VfAKS7 (Figure 2A; Table 1). In addition, we identified AT2G43140, AT4G09180 and AT1G35460 as AKS homologs in *A. thaliana,* and designated them AKS4, AKS5 and AKS6, respectively (Figure 2A). Figure 2B shows a phylogenetic analysis based on the full-length amino acid sequences of AKSs from *V. faba* and *A. thaliana*. VfAKS1 and VfAKS2 were similar to AKS1, and VfAKS3 to AKS3. All possessed two 14-3-3 protein-binding motifs at the N-terminus and middle of the sequence. Previous *in vitro* experiments suggested that phosphorylation of AKS1 at Ser-284, −288 and − 290 induces monomerisation of AKS1 and inhibits its transactivation activity (Takahashi et al., 2017). VfAKS1, VfAKS2, VfAKS3 and AKS3 also possess Ser or Thr upstream of the bHLH domain (Figures 2A,B). In contrast, VfAKS5, AKS5 and AKS6 lack the corresponding Ser or Thr and their sequences were shorter than other AKSs. Of these, VfAKS5 was the shortest and lacked the middle 14-3-3 binding motif. VfAKS4 was similar to AKS2 and AKS4. In contrast, no ortholog of VfAKS6 and VfAKS7 lacking the 14-3-3 protein-binding motif at the N-terminus was found in *A. thaliana*. AKS1, AKS3, AKS5 and AKS6 are identical to FLOWERING BHLH3 (FBH3), FBH4, FBH2 and FBH1, respectively (Ito et al., 2012).

Next, we investigated the expression of VfAKS1–VfAKS7 in GCPs and leaves based on RNA-seq data. The expression levels of VfAKS1, VfAKS3, VfAKS4, VfAKS6 and VfAKS7 were higher in GCPs compared to leaves (Figure 2C). This is consistent with a report that the response is specific to guard cells and is not found in other cell types, such as mesophyll cell protoplasts and root and leaf cells (Takahashi et al., 2007). In addition, previous promoter GUS assay of *AKS1* in *Arabidopsis thaliana* also revealed preferential expression of *AKS1* in guard cells and vascular tissues (Takahashi et al., 2013).

### Identification of Proteins Co-immunoprecipitated With 14-3-3 Protein by MS

ABA-dependent 14-3-3 binding proteins were isolated by co-immunoprecipitation using anti-14-3-3 protein antibodies from Arabidopsis GCPs (Takahashi et al., 2013). Next, we performed immunoprecipitation of ABA-treated *Vicia* GCPs using anti-14-3-3 protein antibodies against *Vicia* 14-3-3a (Emi et al., 2001) and LC–MS/MS using gel segments after resolving the immunoprecipitate by SDS-PAGE. Immunoprecipitates from control and ABA-treated GCPs contained >507 proteins, including those annotated as 14-3-3 proteins, plasma membrane H^+^-ATPases, vacuolar ATP synthase subunit A, pleiotropic drug resistance 12 and clathrin heavy chain (Supplementary Table S1). The number of peptides from VfAKS1–VfAKS5 increased in response to ABA (Table 1).

### The 61 kDa Protein Is the bHLH DNA-Binding Superfamily Protein VfAKS1

**Figure 3:**
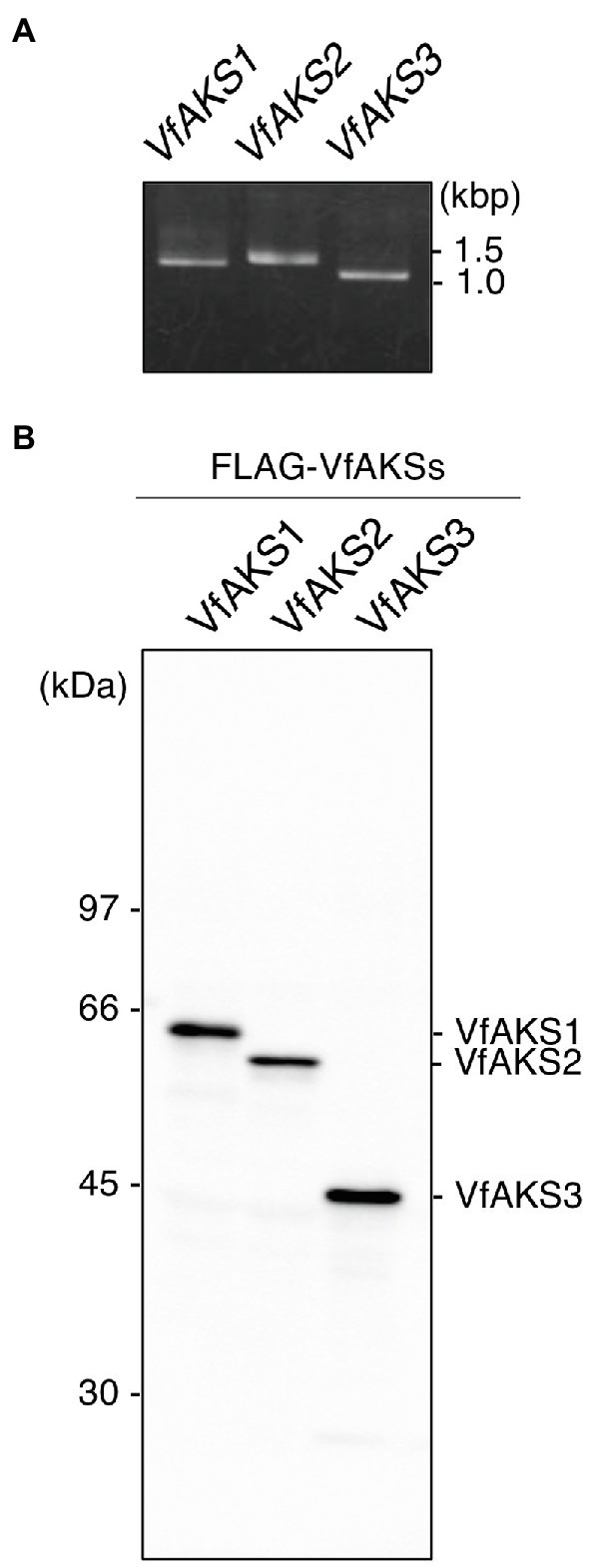
*In vitro* translation of VfAKSs. **(A)**, First PCR of *VfAKSs* for *in vitro* translation. The cording sequences of *VfAKSs* were amplified with adaptor sequences for *in vitro* translation using specific primers. **(B)**, Western blotting of FLAG-VfAKSs. VfAKSs were expressed with a FLAG tag using an *in vitro* translation system and detected with anti-FLAG antibody. Numbers at left indicate molecular weight markers.

VfAKS1–VfAKS5 immunoprecipitated with GCPs, suggesting that one may be the 61 kDa protein. However, the estimated molecular masses of VfAKS1–VfAKS5 based on the amino acid sequences were < 61 kDa (Table 1). Next, we synthesised FLAG-VfAKS1, VfAKS2 and VfAKS3, which had high estimated molecular masses (Table 1), by *in vitro* translation assay and determined their molecular masses by SDS-PAGE. As shown in Figure 3A, full-length CDSs of *VfAKSs* were amplified by RT-PCR from cDNAs of *Vicia* GCPs. Finally, we detected FLAG-VfAKS1 (61.4 kDa), FLAG-VfAKS2 (54.3 kDa) and FLAG-VfAKS3 (43.9 kDa) proteins by Western blotting (Figure 3B). The FLAG tag is around 1.0 kDa. These results indicated that the 61 kDa protein is VfAKS1.

### Phosphoproteomic Analyses Revealed ABA-Dependent Phosphorylation Sites of the VfAKSs

To confirm ABA-dependent phosphorylation of VfAKSs, we performed phosphoproteomic analyses using GCPs treated with ABA. We detected 7,459 phosphopeptides belonging to 2,938 proteins. Of these, 929 phosphopeptides (12.5%) showed at least 2-fold increase in ABA-treated GCPs compared to ABA-untreated GCPs, whereas 489 phosphopeptides (6.6%) showed at least 2-fold decrease.

We detected multiple phosphorylation sites in VfAKS1, VfAKS2, VfAKS3, VfAKS4 and VfAKS6 (Table 2). The conserved Ser residues in the 14-3-3 protein-binding motifs (Figure 2A; Table 2; single asterisk) were phosphorylated in response to ABA (in the case of VfAKS1, Ser-23 and Ser-177). Moreover, ABA-dependent phosphorylation of the conserved Ser or Thr leading to monomerisation and inactivation of transactivation in AKS1 (Takahashi et al., 2016) was observed (Table 2; double asterisks; in the case of VfAKS2, Ser-341 and Thr-345). Ser-182 in VfAKS3 was phosphorylated in response to ABA. Interestingly, Ser-47 of VfAKS1 and Ser-57 of VfAKS2, corresponding to Ser-52 of AKS1, were highly phosphorylated in untreated and ABA-treated GCPs.

**Table 2:** List of phosphorylation sites of VfAKSs detected by phosphoproteomic analyses.

	Phosphosite	PSMs			
		Experiment 1		Experiment 2	
		−ABA	+ABA	−ABA	+ABA
VfAKS1	S23^*^	5	44	8	31
	S47	43	46	51	40
	S63	11	8	20	12
	S177^*^	–	24	–	15
	S294	1	1	–	–
VfAKS2	S33^*^	–	11	–	8
	S57	17	16	13	10
	S341^**^	–	8	–	6
	T345^**^	–	2	–	–
VfAKS3	S19^*^	–	9	–	11
	S19 or S22 (S23)^*^	–	9	–	8
	S22 (or S23)^*^	–	3	–	2
	S182	4	13	4	15
	S275^**^	–	7	–	3
VfAKS4	S33^*^	42	66	41	73
	S225^**^	–	2	–	–
VfAKS6	S266^**^	–	8	–	5
	S266 or S271^**^	–	–	–	1
	S271^**^	–	–	–	3

*PSMs of each phosphopeptides and phosphorylation sites are indicated*.

* *represent the phosphorylation site in 14-3-3 binding sites*.

** *represent the phosphorylation sites corresponding to the sites for monomerisation and inactivation of transactivation in AKS1. –: not detected*.

### Other 14-3-3-Binding Proteins Were Also Phosphorylated in Response to ABA

**Figure 4:**
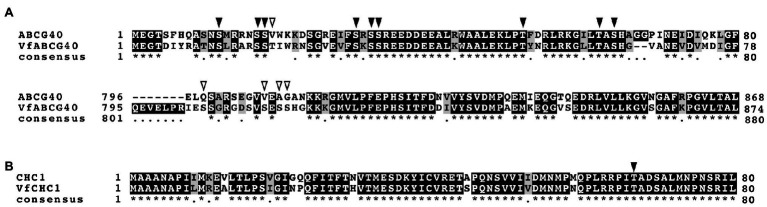
Alignment of ABCG40 and CHC1 from *Vicia faba* and *Arabidopsis thaliana*. **(A)**, Alignment of ABCG40s from *V. faba* (VfABCG40) and *A. thaliana* (AtABCG40) generated with ClustalW (Thompson et al., 1994) using full-length amino acid sequences (VfABCG40 1430 aa and AtABCG40 1423 aa). Black boxes indicate highly conserved residues. Grey box indicates conservative residues. Consensus symbols “^*^” and “.” indicate perfect alignment and a site belonging to a group exhibiting weak similarity, respectively. Phosphorylation sites detected by phosphoproteomic analysis are indicated by arrowheads; black arrowheads, conserved residues in both *A. thaliana* and *V. faba*; and open arrowheads, non-conserved residues. **(B)**, Alignment of CHC1s from *V. faba* (VfCHC1) and *A. thaliana* (AtCHC1) was generated with ClustalW (Thompson et al., 1994) using full-length amino acid sequences (VfCHC1 1701 aa and AtCHC1 1705 aa). Others are the same as **(B)**.

Interestingly, we detected ABA-dependent phosphorylation of proteins annotated as pleiotropic drug resistance 12 (PDR12) and clathrin heavy chain, both of which immunoprecipitated with 14-3-3 protein (Table 3; Supplementary Table S1). Arabidopsis AtPDR12 encodes ATP-binding cassette (ABC) transporter (ABCG40), which is an ABA importer expressed in guard cells. We designated the ortholog in *V. faba* as VfABCG40. VfABCG40 showed high sequence similarity (72%) with AtABCG40. The phosphorylation sites of VfABCG40 detected by phosphoproteomics were located at the N- and C-termini (Figure 4A). Most phospho-Ser and -Thr at the N-terminus were conserved in Arabidopsis ABCG40, but those at the C-terminus were not. At the N-terminus sites, phosphorylation of Ser-30/ Ser-32/ Ser-33 and Thr-62/Ser-64 was slightly upregulated in response to ABA (Table 3).

**Table 3:** List of phosphorylation sites of VfABCG40 and VfCHC1 detected by phosphoproteomic analyses.

	Phosphosite	PSMs			
		Experiment 1		Experiment 2	
		−ABA	+ABA	−ABA	+ABA
VfABCG40	S12	4	–	3	4
	S17 or S18 or T19	23	33	41	43
	S30 or S32 or S33	152	210	121	152
	T51	26	30	20	26
	T62 or S64	71	114	61	81
	S804	1	1	1	1
	S812 or S814 or S815	4	1	2	4
VfCHC1	T67	2	6	2	4

*PSMs of each phosphopeptides and phosphorylation sites are indicated. -: not detected*.

Clathrin heavy chain protein showed high sequence similarity (91%) with Arabidopsis clathrin heavy chain 1 (CHC1; Blackbourn and Jackson, 1996), and we designated its ortholog in *V. faba* as VfCHC1. The phosphorylation site at Thr-67 in VfCHC1 is conserved in AtCHC1 (Figure 4B). ABA induced phosphorylation of Thr-67 (Table 3).

## Discussion

In the present study, RNA-seq-based transcriptome data and targeted 14-3-3 protein-binding protein analyses using highly purified GCPs from *V. faba* revealed that the unknown 61 kDa protein, which is rapidly phosphorylated in response to ABA in *Vicia* guard cells (Takahashi et al., 2007), is a bHLH transcription factor, named *V. faba* ABA-responsive kinase substrate (VfAKS; Figure 2). The AKS family was originally identified in *A. thaliana* as a substrate of ABA-activated OST1/SnRK2 protein kinases (Takahashi et al., 2013; Wang et al., 2013). We identified seven AKS-family genes in *V. faba* and demonstrated rapid ABA-induced AKS phosphorylation and binding to 14-3-3 proteins in guard cells.

VfAKS1 and VfAKS2 had similar SDS-PAGE mobilities to the 61 kDa protein (Figures 1, 3), although their theoretical molecular weights were 47.7 and 48.0 kDa, respectively. The amino acid sequences of these proteins might affect their SDS-PAGE mobilities. Based on the mobility of 61.4 kDa recombinant FLAG-VfAKS1 protein (Figure 3B), VfAKS1 is likely to be the 61 kDa protein of Takahashi et al. (2007). In that study, 43 and 39 kDa proteins were also identified as 14-3-3 protein-binding proteins in response to ABA in *Vicia* guard cells (Takahashi et al., 2007). The molecular mass of FLAG-VfAKS3 by SDS-PAGE was 43.9 kDa, suggesting that the previously reported 43 kDa protein is VfAKS3.

MS identified *in vivo* ABA-dependent phosphorylation sites of VfAKS-family members, including VfAKS2 Ser-341 and Thr-345 (Table 2), corresponding to Arabidopsis AKS1 (AtAKS1) Ser-284 and -288. *In vitro*, AtAKS1 phosphorylation at these serine residues is mediated by OST1/SnRK2.6 protein kinase and inhibits DNA binding by AtAKS1 by disrupting AtAKS1 dimer formation (Takahashi et al., 2016). This may be the mechanism by which ABA represses the expression of genes, including Arabidopsis *KAT1* inward-rectifying potassium channel, which functions in stomatal opening in guard cells (Takahashi et al., 2017). However, it is unclear whether these phosphorylations are induced in response to ABA in plant cells. Our results provide *in vivo* evidence supporting the proposed model of phosphorylation-dependent control of AKS transcription factor function (Table 2). In addition, amino acid alignment analyses suggest that these phosphorylation sites are conserved in many AKS-family members in *A. thaliana* and *V. faba* (Figure 2A). Note that we did not detect phosphorylation of VfAKS1 at Ser-320 and Thr-324, corresponding to AtAKS1 Ser-284 and Ser-288, despite sufficient phosphopeptides from the 14-3-3 binding site (Table 2). Moreover, phosphoproteomic analyses revealed new phosphorylation residues in GCPs from *V. faba*. Ser-47 of VfAKS1 and Ser-57 of VfAKS2, phosphorylation of which was detected in control and ABA-treated GCPs, were conserved in AKS1 (Table 2; Figure 2A). Other physiological signals may regulate phosphorylation of these residues.

In this study, we identified >500 proteins that co-immunoprecipitated with 14-3-3 proteins in GCPs from *V. faba*. Of them, orthologs of Arabidopsis ABCG40 and CHC1 in *V. faba* were detected as phosphorylated proteins by phosphoproteomic analysis. Arabidopsis ABCG40 expressed in stomatal guard cells imports ABA, synthesised in the vascular bundle in response to drought stress, into guard cells, leading to stomatal closure (Kang et al., 2010). In this study, ABA-dependent phosphorylation of ABCG40 at Ser-30/Ser-32/Ser-33 and Thr-62/Ser-64, corresponding to Ser-30/Ser-32/Ser-33 and Thr-62/Ser-64 in AtABCG40, was slightly upregulated in response to ABA in 10 min (Figure 4; Table 3). The function of CHC1 in stomatal movement has been investigated using *A. thaliana*. Plessis et al. (2011) isolated a *hot ABA-deficient suppressor 1* (*has1*) suppressor mutant of *ABA deficient3* (*aba3-1*) by infrared imaging. The *has1* mutant showed a closed stomatal phenotype in response to a low concentration of exogenous ABA and drought tolerance. Larson et al. (2017) identified *CHC1* as responsible for *has1* mutant and found that *chc1* and *chc2* affect endocytosis, exocytosis and stomatal movement. To our knowledge, this is the first report that CHC1 is phosphorylated in response to ABA. Further investigation is needed of the roles of ABA-dependent phosphorylation of ABCG40 and CHC1.

*V. faba* is used for research on stomatal movement (Willmer and Fricker, 1996). However, the limited genomic information on *V. faba* because of its large genome (13 Gb; Kaur et al., 2012) had hampered identification of the 61 kDa protein in *Vicia* GCPs by MS (Takahashi et al., 2007). We constructed an expression database of *V. faba* from RNA-seq data and identified VfAKS1 as the 61 kDa protein. Furthermore, the database allows phosphoproteomic analysis of *Vicia* GCPs in response to various signals.

## Data Availability Statement

The datasets presented in this study can be found in online repositories. The names of the repository/repositories and accession number(s) can be found at: http://www.proteomexchange.org/, PXD027057 and PXD027058, and DNA Data Bank of Japan (https://www.ddbj.nig.ac.jp/index-e.html) under accession number DRA012337.

## Author Contributions

YH, YoT, and TK designed the experiments and wrote the manuscript. YH, YoT, KF, YaT, KT, KK, TS, and TK performed the experiments. All authors reviewed the manuscript.

## Funding

This research was supported by the Grants-in-Aid for Scientific Research from MEXT (15H05956, 20H05687 and 20H05910 to TK) and PRESTO, Japan Science and Technology Agency (Grant Number JPMJPR21D8 to YoT).

## Conflict of Interest

The authors declare that the research was conducted in the absence of any commercial or financial relationships that could be construed as a potential conflict of interest.

## Publisher’s Note

All claims expressed in this article are solely those of the authors and do not necessarily represent those of their affiliated organizations, or those of the publisher, the editors and the reviewers. Any product that may be evaluated in this article, or claim that may be made by its manufacturer, is not guaranteed or endorsed by the publisher.
