# Supplementary material for: Identification of Abscisic Acid-Dependent Phosphorylated Basic Helix-Loop-Helix Transcription Factors in Guard Cells of Vicia faba by Mass Spectrometry
Source: Front Plant Sci. 2021 Dec 20;12:735271. doi: 10.3389/fpls.2021.735271 (PMC8721282; doi:10.3389/fpls.2021.735271)
Supplement: Supplementary file 1 [file Table_1.docx]

**TABLE S1**. List of proteins in immunoprecipitation using anti-14-3-3 protein antibody.

| Accession | Description | Experiment 1 | | Experiment 2 | | Experiment 3 | |
| --- | --- | --- | --- | --- | --- | --- | --- |
|  |  | -ABA | +ABA | -ABA | +ABA | -ABA | ABA |
| Vfab029609 | 14-3-3 OMEGA | 1327 | 1422 | 1505 | 1472 | 191 | 183 |
| Vfab006708 | H(+)-ATPase 1 | 749 | 593 | 689 | 635 | 506 | 414 |
| Vfab014126 | H(+)-ATPase 1 | 453 | 368 | 380 | 376 | 283 | 219 |
| Vfab006556 | 14-3-3 OMEGA | 383 | 372 | 398 | 377 | 50 | 53 |
| Vfab027176 | H(+)-ATPase 11 | 351 | 283 | 308 | 283 | 254 | 192 |
| Vfab029659 | vacuolar ATP synthase subunit A | 263 | 179 | 177 | 217 | 46 | 28 |
| Vfab029667 | 14-3-3 LAMBDA | 234 | 190 | 181 | 182 | 52 | 53 |
| Vfab029501 | pleiotropic drug resistance 12 | 195 | 156 | 213 | 199 | 80 | 59 |
| Vfab020562 | cytosolic invertase 2 | 184 | 238 | 259 | 250 | 54 | 49 |
| Vfab019071 | H(+)-ATPase 5 | 175 | 145 | 157 | 166 | 129 | 101 |
| Vfab029514 | 14-3-3 IOTA | 173 | 140 | 125 | 143 | 32 | 31 |
| Vfab028816 | Clathrin, heavy chain | 161 | 120 | 185 | 140 | 22 | 12 |

Top 12 of 507 coprecipitated proteins are listed. Peptide spectrum matches (PSMs), the total number of identified peptide spectra matched for the protein, of each protein are indicated. Description represents the functional annotation of contigs by BLASTX analysis against Arabidopsis database.
