# Supplementary material for: Identification of Abscisic Acid-Dependent Phosphorylated Basic Helix-Loop-Helix Transcription Factors in Guard Cells of Vicia faba by Mass Spectrometry
Source: Front Plant Sci. 2021 Dec 20;12:735271. doi: 10.3389/fpls.2021.735271 (PMC8721282; doi:10.3389/fpls.2021.735271)
Supplement: Supplementary file 2 [file Table_2.docx]

**TABLE S2**. List of primers for 1st PCR of *in vitro* transcription

|  |  | 5’ to 3’ |
| --- | --- | --- |
| *VfAKS1* | Fw | CCAGCAGGGAGGTACTATGGAGTCAGATCTTCATCAACAGC |
| *VfAKS1* | Rv | CCTTATGGCCGGATCCAAGAGCTCTTTTTTTTTTTTATTGTTGCTTCTTGTGTGGGC |
| *VfAKS2* | Fw | CCAGCAGGGAGGTACTATGGAGTCAGATCTTCAGCAGC |
| *VfAKS2* | Rv | CCTTATGGCCGGATCCAAGAGCTCTTTTTTTTTTTTATTGGCGATGAGAACACGTG |
| *VfAKS3* | Fw | CCAGCAGGGAGGTACTATGGATTCCAATACTCAACAGAACC |
| *VfAKS3* | Rv | CCTTATGGCCGGATCCAAGAGCTCTTTTTTTTTTTTAAGGAATTTGATTTGTATCTT  CCTTCTGC |
